# Taxonomic placement of *Paphiopedilum rungsuriyanum* (Cypripedioideae; Orchidaceae) based on morphological, cytological and molecular analyses

**DOI:** 10.1186/s40529-017-0170-1

**Published:** 2017-03-29

**Authors:** Yung-I Lee, Mei-Chu Chung, Kongmany Sydara, Onevilay Souliya, Sulivong Luang Aphay

**Affiliations:** 1 Department of Biology, National Museum of Natural Science, No 1, Kuan-Chien Rd, Taichung, 40453 Taiwan, ROC; 2 Department of Life Sciences, National Chung Hsing University, No 145, Xingda Rd, Taichung, 40227 Taiwan, ROC; 3 Institute of Plant and Microbial Biology, Academia Sinica, No 128, Sec. 2, Academia Rd, Nankang, Taipei, 11529 Taiwan, ROC; 4 Institute of Traditional Medicine, Ministry of Health, Phonepapao Village, Sisattanack District, 856 Vientiane, Lao People’s Democratic Republic; 5 Luangaphay Incorporation Sole Co., Ltd, 034 Phonsinouan Road, Dongpalane Thong Village, Sisattanak District, 1000 Vientiane, Lao People’s Democratic Republic

**Keywords:** Infrageneric classification, Karyotype, Morphology, Molecular phylogeny, *Paphiopedilum*

## Abstract

**Background:**

*Paphiopedilum rungsuriyanum* from Northern Laos was discovered and described in 2014. It is characterized by having miniature tessellated leaves, a flower having a helmet shaped lip with a V-shaped neckline, and a semi-lunate, 3-dentate staminode with an umbo. These morphological features distinguish *P. rungsuriyanum* from the other known sections/subgenera of *Paphiopedilum*, making it difficult to group with existing infrageneric units.

**Results:**

*Paphiopedilum rungsuriyanum* has chromosome number of 2n = 26. Fluorescence in situ hybridization study demonstrates that there are two 45S rDNA signals in the telomeric region of chromosomes, and more than 20 5S rDNA signals dispersed signals in the pericentromeric and centromeric regions. Phylogenetic analyses based on four nuclear (i.e. ITS, *ACO*, *DEF*4 and *RAD*51) and four plastid (i.e. *atp*I-*atp*H, *mat*K, *trn*S-*trn*fM and *ycf*1) gene regions indicate that *P. rungsuriyanum* is nested in subgenus *Paphiopedilum* and is a sister to section *Paphiopedilum*.

**Conclusions:**

The results in combination with karyomorphological, rDNA FISH patterns, morphological and phylogenetic analyses suggest a new section *Laosianum* to accommodate this species in the current sectional circumscription of subgenus *Paphiopedilum*.

**Electronic supplementary material:**

The online version of this article (doi:10.1186/s40529-017-0170-1) contains supplementary material, which is available to authorized users.

## Background

The genus *Paphiopedilum* comprises about 80 species, extending from the Himalayas and southern China through Malaysia to Guadalcanal (Braem 1988; Cribb 1998). The beautiful flowers of *Paphiopedilum* species are shaped like the slipper of Aphrodite and hold a place in the affections of orchid hobbyists in the world. In the wild, *Paphiopedilum* populations are found in relatively restricted areas, and most *Paphiopedilum* species are endangered or even facing extinction because of the over-collection and the destruction of their habitats. *Paphiopedilum* can be classified into three subgenera including subgenus *Parvisepalum*, subgenus *Brachypetalum* and subgenus *Paphiopedilum* based on morphological, cytological and molecular phylogenetic data (Cribb 1998; Chochai et al. 2012). In addition, the subgenus *Paphiopedilum* could be divided into five sections: *Paphiopedilum*, *Barbata*, *Cochlopetalum*, *Coryopedilum* and *Pardalopetalum*.

The limestone mountains of Indochina are home to a great diversity of endangered *Paphiopedilum* species. During the past two decades, a number of amazing *Paphiopedilum* species with very limited distribution were discovered in this area, such as *P. vietnamense* and *P. hangianum* (Averyanov et al. 2003; Liu et al. 2009). For now, the central region of the Indochina, particularly the territory of Laos, contains the largest part of the Indochinese limestone mountain which remains to be investigated. These inaccessible areas, undoubtedly, are home for numerous unknown plant species, particularly for strictly endemic orchids. A few years ago, *P. canhii* was discovered and described from the limestone mountain in north-western Vietnam near the Laotian border (Averyanov 2010; Averyanov et al. 2010). The distinct flower morphology led some taxonomists to propose a new section *Pygmaea* under subgenus *Paphiopedilum* (Averyanov et al. 2011), or even a new subgenus *Megastaminodium* (Braem and Gruss 2011) to accommodate this species. Afterward, based on the cytological data, phylogenetic analyses using plastid and nuclear genes and morphological characters, Gorniak et al. (2014) suggested the status of the separate subgenus *Megastaminodium* within the genus *Paphiopedilum* as proposed by Braem and Gruss (2011). In 2014, *P. rungsuriyanum* was identified as a new species from smuggled plants under the name of *P. canhii* from Laos (Gruss et al. 2014). Later, more plants were found on the rocky limestone in Northern Laos. Although the tiny plants with marbled leaves look similar to *P. canhii*, the other morphological characteristics of flower, such as staminodial shield, lip, and petal/sepal ratio and color are distinct from *P. canhii* and species in the other sections/subgenera. Therefore, more detail studies are required when we are going to propose the taxonomic status to accommodate *P. rungsuriyanum*.

*Paphiopedilum* has been characterized by the significant chromosome variation, ranging from 2n = 26 to 42 (Duncan and Macleod 1949; Karasawa 1979; Karasawa and Aoyama 1988). The changes of chromosome number and karyotype are suggested to be caused by Robertsonian translocation, e.g. the fission of metacentric chromosomes at the centromeric region to generate more telocentric chromosomes (Karasawa and Saito 1982; Jones 1998). In addition, results from FISH mapping of ribosomal rRNA genes indicates that the duplication of 25S rDNA loci occurred independently in subgenus *Parvisepalum* and the sections *Coryopedilum* and *Pardalopetalum* of subgenus *Paphiopedilum*, while the duplication of 5S rDNA loci can be detected only in subgenus *Paphiopedilum* (Lee and Chung 2008; Lan and Albert 2011). Together, these data (in combination of chromosome number, karyotype and rDNA site) provide valuable information for cytotaxonomy in the subgenus/section level of *Paphiopedilum*. This study aims to provide cytological, molecular and morphological data which could cast new light on the discussion on the taxonomic position of *P. rungsuriyanum* within the genus.

## Methods

### Plant material

The materials of *P. rungsuriyanum* and *P. canhii* were obtained from the orchid collection of Sulivong Luang Aphay in Lao PDR. The samples are accompanied by CITES permits. Voucher and GenBank accession numbers are listed in Additional file 1: Table S1.

### Chromosome preparation

Root tips of *Paphiopedilum* species were harvested and pretreated in 2 mM 8-hydroxyquinoline at 20 °C for 5 h. After being rinsed with distilled water, the root tips were then fixed in fresh prepared Farmer’s fluid (three parts of ethanol to one part of glacial acetic acid). Root tips were macerated with 6% cellulose (Onozuka R-10, Yakult Honsha, Tokyo, Japan) and 6% pectinase (Sigma Chemical Co.) in 75 mM KCl, pH 4.0 at 37 °C for 90 min, and stained in 2% aceto-orcein, and then squashed on slide according to the methods of Karasawa and Aoyama (1988). The images of well-spread chromosome complements were captured by a CCD camera attached to a light microscope (Axioskop 2, Carl Zeiss AG, Germany). For the subsequent FISH experiments, the cover glasses were removed with a razor blade after freezing the slide in liquid nitrogen. Slides were dried and stored at −80 °C until required.

### Fluorescence in situ hybridization (FISH)

The FISH procedure was essentially the same as previously described protocols (Lee et al. 2011). The rDNA probes used in this study were 45S rDNA (pTA71) from *Triticum aestivum* (Gerlach and Bedbrook 1979) and 5S rDNA (pTA794) from *T. aestivum* (Gerlach and Dyer 1980). All probes were labeled by nick translation with digoxigenin-11-dUTP or biotin-16-dUTP (Roche Apply Science, Basel, Switzerland). The digoxigenin-labeled probes were detected by anti digoxigenin–rhodamine (Roche Apply Science). The biotin-labeled probes were detected by anti-biotin-fluorescein isothiocyanate (FITC) (Vector Laboratories, Burlingame, CA, USA). Chromosomes were counterstained with 4′,6-diamidino-2-phenylindole (DAPI) in an antifade solution (Vector Laboratories, CA, USA). Images were taken by a CCD camera attached to an epifluorescence microscope (Axioskop 2, Carl Zeiss AG, Germany).

### DNA extraction, amplification and sequencing

Total DNA was extracted from silica-gel dried leaf by using DNeasy Plant Mini Kit (Qiagen, Hilden, Germany) according to the manufacturer’s protocols. The nuclear ribosomal spacer regions, ITS1 and ITS2, and the 5.8S ribosomal gene (ITS) (Douzery et al. 1999), three low-copy nuclear genes (*ACO*, *DEF*4 and *RAD*51) and four plastid regions (*atp*I-*atp*H, *matK*, *trn*S-*trn*fM and *ycf*1) were amplified with the same primers as in Guo et al. (2015) and listed in Additional file 2: Table S2. PCR amplification and the Sanger sequencing were carried out as described by Guo et al. (2015). PCR products that were difficult to sequence directly were cloned using the pGEM-T Vector System II (Promega, Madison, WI, USA). The sequencing reactions were performed by the DNA analysis core laboratory (Institute of Plant and Microbial Biology, Taipei, Taiwan).

### Phylogenetic analyses

Sequences were identified (Additional file 1: Table S1) using a BLAST search against the NCBI sequence database (National Center for Biotechnology Information, GenBank) to find the closest sequence matches in the database. For phylogenetic analysis, four low-copy nuclear genes (*ACO*, *DEF*4, ITS and *RAD*51), four plastid regions (*atp*I-*atp*H, *matK*, *trn*S-*trn*fM and *ycf*1) and the combined all data were added to the analysis by referring the data matrices created by Guo et al. (2015), and sequences of *Mexipedium xerophyticum* and *Phragmipedium longifolium* were used as outgroup taxa. DNA sequences were aligned using CLUSTALX (Thompson et al. 1997), followed by manual adjustment. The alignment matrices were analyzed by the maximum parsimony (MP) using PAUP* version 4.0b10 (Swofford 2003) with tree-bisection-reconnection (TBR) branch swapping and the MULTREES (holding multiple trees) option in effect with 1000 replicates of random sequence addition. Tree length, consistency index (CI) and retention index (RI), and ts/tv ratio were calculated. Support for groups was evaluated using the bootstrap method (Felsenstein 1985) with 1000 replicates. For checking the incongruence between plastid and nuclear data, the incongruence length difference (ILD) test (Farris et al. 1994) was performed in PAUP* version 4.0b10, and 1000 replicates with the same settings as in the heuristic searches were conducted.

Phylogenetic relationships were further analyzed by a model-based Bayesian approach using MrBayes 3.2.1 (Ronquist and Huelsenbeck 2003). The best-fitting model of evolution (Additional file 3: Table S3) was selected under Akaike information criterion test (Akaike 1974) as implemented in MrModeltest 2.2 (Nylander 2004). Two separate runs of four Monte Carlo Markov chains (MCMC) (Yang and Rannala 1997) were run for 3,000,000 generations until the mean deviation of split frequency dropped below 0.01, and a tree was sampled every 1000th generation. Trees from the first 25% of generations were discarded using the “burn-in” command, and the remaining trees were used to calculate a 50% majority-rule consensus topology and to determine PP for individual branches. The trees obtained in these analyses were drawn with the TreeGraph 2 software (Stover and Muller 2010).

## Results

### Morphological characters

**Fig. 1:**
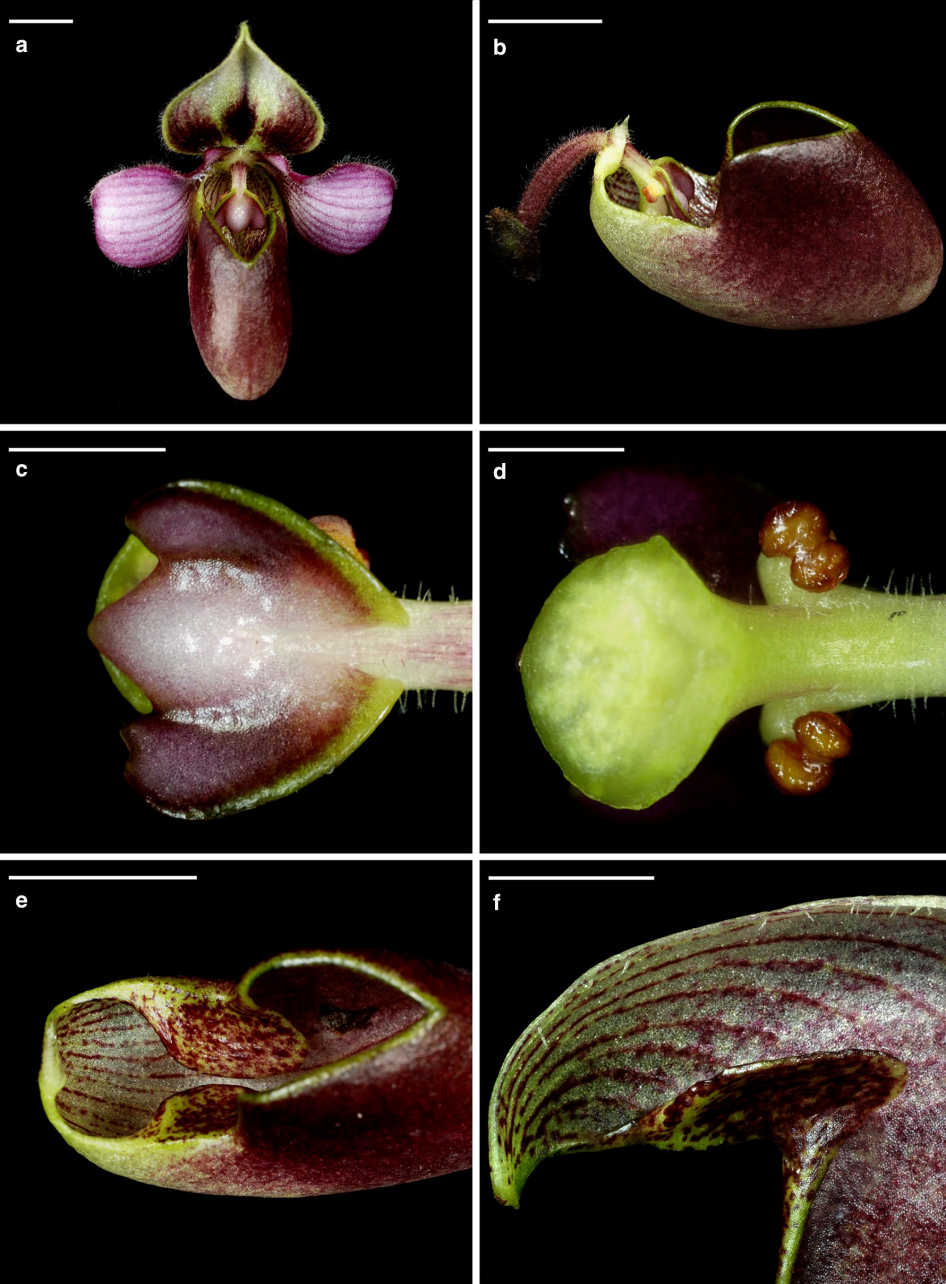
Morphological features of *P. rungsuriyanum*. **a** Flower. *Scale bar* 10 mm. **b** The lateral view of lip, staminode and ovary. *Scale bar* 10 mm. **c** The staminode. *Scale bar* 5 mm. **d** The smooth stigmatic surface. *Scale bar* 2 mm. **e** Lip. The visible incurved smooth lateral lobes. *Scale bar* 10 mm. **f** The trichome arrangement. *Scale bar* 5 mm

The floral structure, including column (staminode and stigma), lip, sepals and petals provides valuable taxonomic traits in *Paphiopedilum* classification (Atwood 1984; Braem 1988; Cribb 1998). The miniature plant of *P. rungsuriyanum* with its marbled leaves looks similar to *P. canhii*, nevertheless their flowers differ significantly from each other in terms of flower colors, petal and lip shapes, and the shape of staminode (Fig. 1; see the plates in Gorniak et al. 2014). The staminode of *P. rungsuriyanum* is distinct from *P. canhii*, being half-moon shaped with three lobes and a clear bulge in the middle (Fig. 1c), while the staminode of *P. canhii* has an ovate-elliptic shape that is entire and unlobed. The stigmatic surface of *P. rungsuriyanum* is smooth and its pollinium is viscid (Fig. 1d), which are the same as *P. canhii* and most *Paphiopedilum* species (except for species of subgenus *Parvisepalum*, having mammillate stigmatic surface and granular pollinia).

**Fig. 2:**
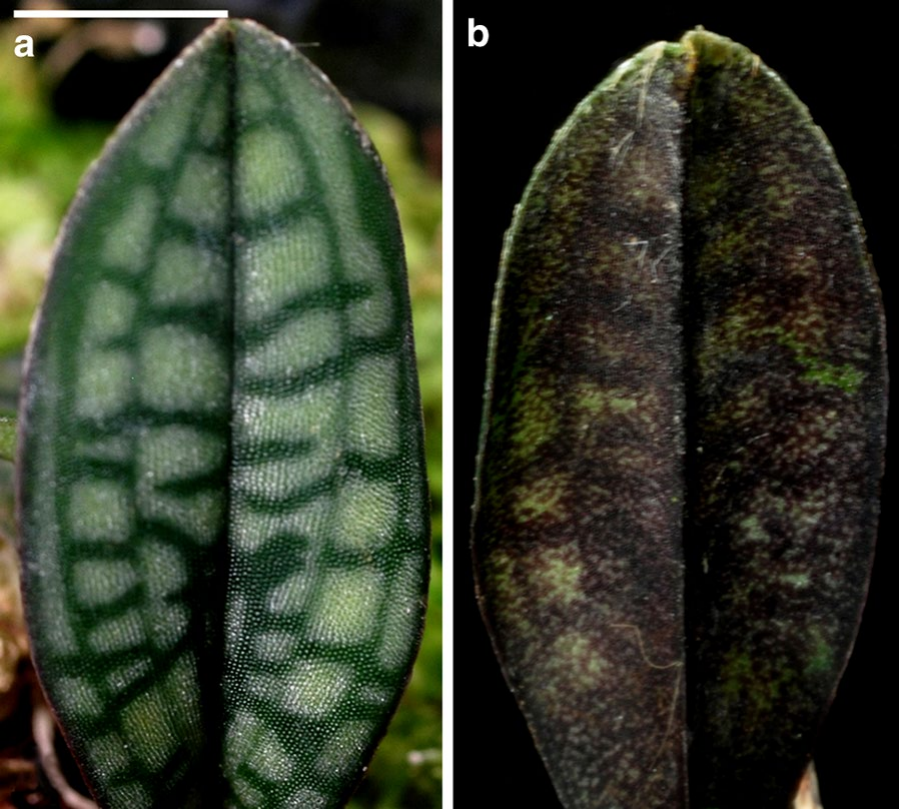
Leaf morphology of *P. rungsuriyanum*. **a** The adaxial surface of leaves. *Scale bar* 5 mm. **b** The abaxial surface of leaves. *Scale bar* 5 mm

The lip of *P. rungsuriyanum* is different from that of *P. canhii*, being helmet shaped with incurved lateral lobes (Fig. 1a, b, e). There are several red–purple spots on lateral lobes. The outer surface of lip of *P. rungsuriyanum* is smooth, while the inner surface has a few trichomes (Fig. 1f). As compared with *P. canhii*, the trichomes of *P. rungsuriyanum* lip are shorter and less dense. The petal of *P. rungsuriyanum* is characterized by its oval shape and intensively red–purple veins (Fig. 1a). The margin of petal has whitish translucent trichomes. The margins and abaxial side of the dorsal sepal and the synsepal are densely pubescent. Besides, the pedicel, ovary and peduncle are also pubescent, covered by trichomes (Fig. 1b). *P. rungsuriyanum* has marbled leaves with a regular pattern of brighter blotches (Fig. 2).

### Cytological data

**Fig. 3:**
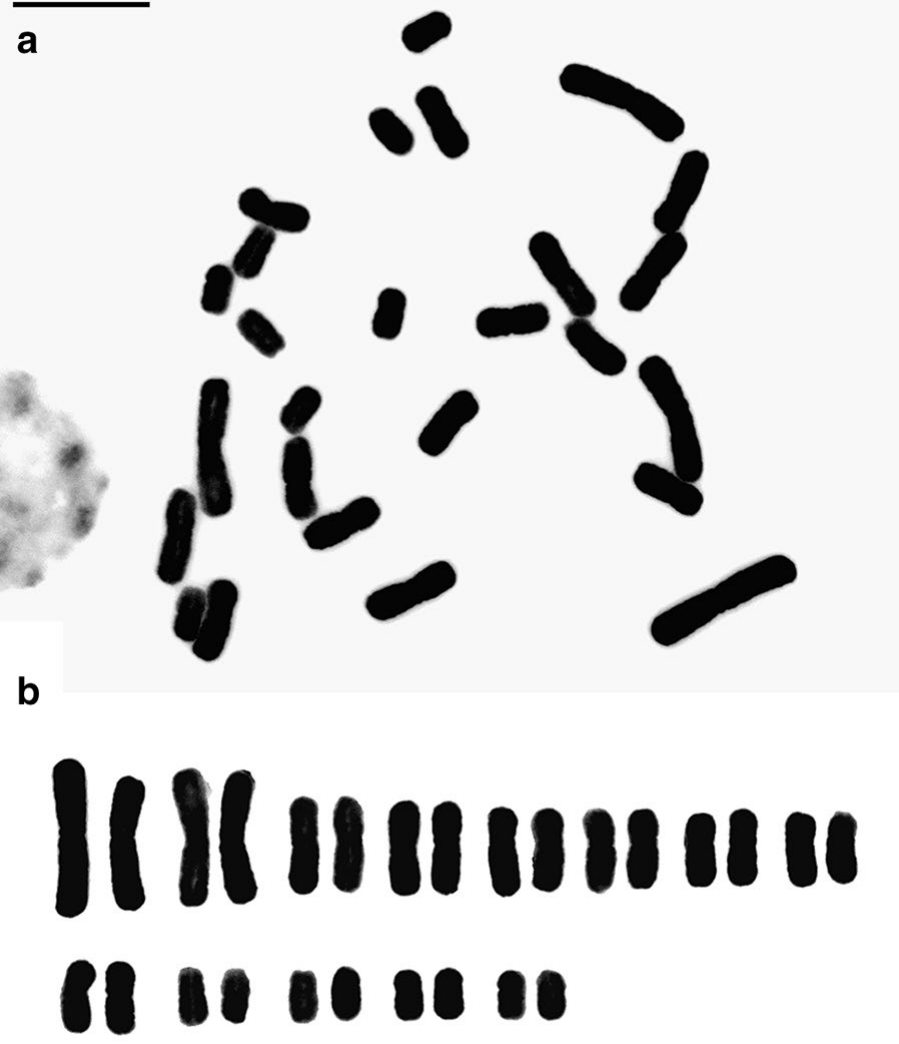
Karyomorphology of *P. rungsuriyanum*. **a** Mitotic chromosome preparation, 2n = 26. *Scale bar* 10 μm. **b** Karyotype arrangement. *Scale bar* 10 μm

The chromosome number of 2n = 26 is counted here for the first time for *P. rungsuriyanum*. The chromosome complement is constituted from four large chromosomes varying in length from 12.1 to 10.0 μm, and 22 small chromosomes varying from 7.2 to 3.8 μm, showing a distinct bimodal karyotype. All chromosomes are median type with arm ratios ranging from 1.0 to 1.4 (Fig. 3).

### Distribution patterns of ribosomal DNA by FISH (rDNA-FISH)

**Fig. 4:**
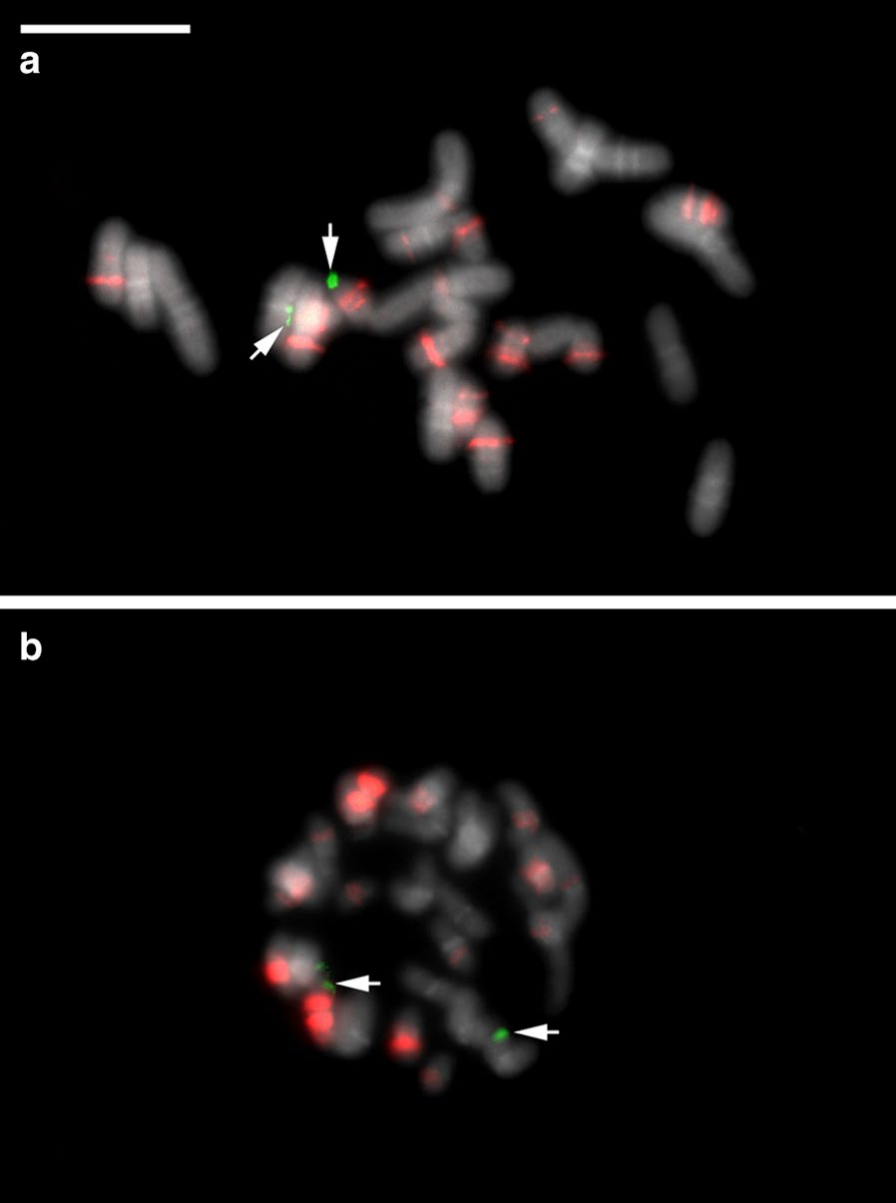
Two-colored FISH of 5S rDNA and 45S rDNA on metaphase chromosomes. **a** *P. rungsuriyanum*. **b** *P. canhii*. Chromosomes were counterstained with DAPI, and 45S rDNA (*green*) and 5S rDNA (*red*) sites were simultaneously detected in one reaction. In *P. rungsuriyanum*, 45S rDNA signals (*arrows*) and 5S rDNA signals were detected on the same chromosomes, while in *P. canhii*, 45S rDNA signals (*arrows*) and 5S rDNA signals were separated on different chromosomes. *Scale bar* 10 μm

The rDNA-FISH results show two chromosomes of *P. rungsuriyanum* with 45S rDNA signals in the telomeric region. Two 5S rDNA sites were present on the chromosomes bearing 45S rDNA sites, and 20 more dispersed signals in the pericentromeric and centromeric regions (Table 1; Fig. 4a). In *P. canhii*, there are two 45S rDNA signals in the telomeric region, and at least eight major 5S rDNA signals and about 12 dispersed repeats in pericentromeric and centromeric regions (Table 1; Fig. 4b).

**Table 1:** The diploid chromosome numbers and rDNA FISH patterns of *Paphiopedilum* subgenera/sections

Subgenus/section	2n	Number of rDNA sites^a^	
		25S	5S
Subgenus *Parvisepalum*	26	2–4	2
Subgenus *Brachypetalum*	26	2	2
Subgenus *Paphiopedilum*			
Section *Laosianum*	26	2	20
Section *Megastaminodium*	26	2	22
Section *Paphiopedilum*	26, 30	2	14–21
Section *Coryopedilum*	26	2–9	16–32
Section *Pardalopetalum*	26	2–6	8–34
Section *Barbata*	32–42	2	2–18
Section *Cochlopetalum*	30–38	2	20–25

^a^The rDNA FISH patterns at subgenus/section-level (except for sections *Laosianum* and *Megastaminodium*) published by Lan and Albert (2011)

### Molecular analyses

**Fig. 5:**
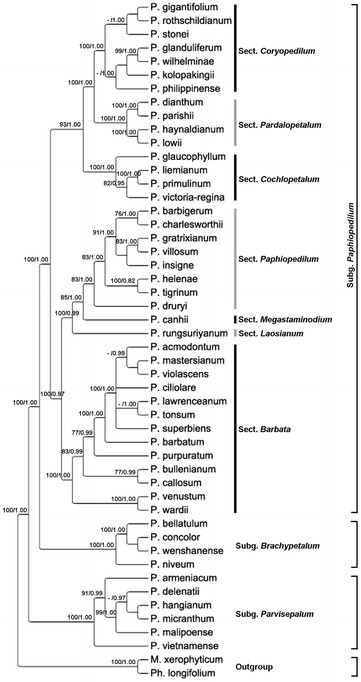
One of the most parsimonious trees from the combined analysis of ITS, three low-copy nuclear genes (*ACO*, *DEF*4 and *RAD*51) and four plastid regions (*atp*I-*atp*H, *matK*, *trn*S-*trn*fM and *ycf*1) for *Paphiopedilum*. Bootstrap percentages (BP) >70 and Bayesian posterior probabilities (PP) are given for supported clades above the branches

The analyses of nuclear genes (i.e. ITS, *ACO*, *DEF*4 and *RAD*51 data matrixes), plastid data matrix and combined data matrix were demonstrated by one of the most parsimonious trees (see Fig. 5; Additional file 4: Figure S1, Additional file 5: Figure S2, Additional file 6: Figure S3, Additional file 7: Figure S4, Additional file 8: Figure S5). Statistics of taxa number, including positions in matrix, variable site, parsimony-informative sites, tree length, consistency index (CI) and retention index (RI) for one of the most parsimonious trees from each analysis is shown in Table 2. Tree topology, bootstrap percentages, branches that collapse in the strict consensus tree obtained from maximum parsimony analysis and Bayesian posterior probability values are indicated in Fig. 5.

**Table 2:** Parsimony statistics for phylogenetic analyses from single and combined datasets

Matrix	ITS	*ACO*	*DEF*4	*RAD*51	Combined plastid	Combined
Number of taxa	50	50	48	50	50	50
Included positions in matrix	734	1784	1131	931	3782	8362
Variable site	340	723	316	702	632	2713
Parsimony-informative sites	180	364	166	195	262	1167
Tree length	574	1231	417	1050	813	4237
Consistency index (CI)	0.794	0.77	0.868	0.851	0.84	0.789
Retention index (RI)	0.847	0.816	0.93	0.785	0.871	0.815

In the analysis of combined data matrix, the *Paphiopedilum* species form a strongly supported monophyletic group (100 BS, 1.00 PP), and the division of the genus *Paphiopedilum* into three subgenera, i.e. subgenera *Parvisepalum*, *Brachypetalum* and *Paphiopedilum* is well supported (100 BP, 1.00 PP for all). The position of *P. rungsuriyanum* on the multicopy nuclear ITS, nuclear low copy genes, i.e. *ACO*, *DEF*4, *RAD*51, and plastid data remain in conflict. According to the ITS data, *P. rungsuriyanum* is sister to species of the section *Paphiopedilum* (Additional file 4: Figure S1). However, on the *ACO*-based tree, *P. rungsuriyanum* is sister to the subgenus *Paphiopedilum* (Additional file 5: Figure S2); on the *DEF*4-based tree, *P. rungsuriyanum* is embedded in the section *Paphiopedilum* (Additional file 6: Figure S3); on the *RAD*51-based tree, *P. rungsuriyanum* is sister to the clade comprising species of sections *Paphiopedilum*, *Barbata*, *Coryopedilum* and *Pardalopetalum* (Additional file 7: Figure S4). According to the combined plastid matrix, *P. rungsuriyanum* is embedded in the clade comprising sections *Barbata* and *Paphiopedilum* (Additional file 8: Figure S5). The analysis of combined data matrix indicates that *P. rungsuriyanum* is sister species of the section *Paphiopedilum*, and is deeply embedded in the subgenus *Paphiopedilum* (Fig. 5).

## Discussion

To assess taxonomic position of *P. rungsuriyanum* within the genus *Paphiopedilum*, we compared the cytological, molecular and morphological data obtained from the representative species of each subgenus in *Paphiopedilum* according to the report by Gorniak et al. (2014). Furthermore, we investigated the distribution patterns of rDNA signals in *P. rungsuriyanum* and *P. canhii* for cytotaxonomic reference. The major significant characters among the subgroups are summarized in Table 2.

### Cytological and rDNA FISH analysis

Previous cytological studies on *Paphiopedilum* species have provided valuable data for cytotaxonomy (Karasawa and Saito 1982). In *Paphiopedilum*, the diploid chromosome number ranges from 2n = 26 (all metacentric chromosomes) to 2n = 42 [with the conserved arm number (n.f.) = 52]. Species of subgenera *Parvisepalum* and *Brachypetalum*, and the three sections of the subgenus *Paphiopedilum*, i.e. *Paphiopedilum* (except *P. druryi* and *P. spicerianum* with 2n = 30) *Coryopedilum* and *Pardalopetalum* possess 2n = 26, while two other sections of the subgenus *Paphiopedilum*, i.e. *Barbata* and *Cochlopetalum*, have the chromosome complement of 2n = 28–42 (n.f. = 52–54) and 2n = 30–37 (n.f. = 50), respectively. In section *Cochlopetalum*, their common ancestor might lose either two telocentric chromosomes or a single metacentric chromosome before divergence of extant species (Cox et al. 1998). Phylogenetic analyses have indicated that plesiomorphic karyotype for *Paphiopedilum* possessed 26 metacentric chromosomes with increases in chromosome number accomplished by centric fission (Cox et al. 1997, 1998). In our karyotype analysis, *P. rungsuriyanum* has the chromosome complement of 2n = 26 (Fig. 3), belonging to the groups with plesiomorphic karyotype. Therefore, we may exclude *P. rungsuriyanum* as a member of sections *Barbata* and *Cochlopetalum*.

In *Paphiopedilum*, the numbers and distribution patterns of rDNA loci exhibit a considerable diversity that correlates well with phylogenetic lineages and provide important markers for cytotaxonomy (Lan and Albert 2011). The most parsimonious ancestral number of 25S rDNA sites in *Paphiopedilum* is two, and duplication of 25S rDNA loci could be detected in subgenus *Parvisepalum* and in sections *Coryopedilum* and *Pardalopetalum* of subgenus *Paphiopedilum*. Massive duplication event of 5S rDNA loci occurred in all five sections of subgenus *Paphiopedilum*, while the early diverging subgenera, i.e. *Parvisepalum* and *Brachypetalum* retained two 5S rDNA sites (Table 2). In this study, both *P. rungsuriyanum* and *P. canhii* possess only two 45S rDNA sites and significant duplication of 5S rDNA sites (Fig. 4a, b). In *P. rungsuriyanum* but not *P. canhii*, one of the major 5S rDNA signals are closely linked with the 45S array that is similar to the pattern of rDNA signals in section *Paphiopedilum*. From the rDNA FISH data, we may exclude *P. rungsuriyanum* as a member of subgenera *Parvisepalum* and *Brachypetalum* and suggest a closer relationship to subgenus *Paphiopedilum*.

### Comparative analysis of molecular and morphological data

*Paphiopedilum rungsuriyanum* and *P. canhii* are found in the limestone areas in Laos. Although both of them have the miniature plants with tessellated leaves and the chromosome number of 26, their flowers are clearly different and distinct from species in the other subgenera/sections (see Additional file 9: Table S4). A new subgenus *Megastaminodium* (Braem and Gruss 2011) or a new section *Pygmaea* (Averyanov et al. 2011) has been proposed to accommodate *P. canhii*, but it now looks difficult to place *P. rungsuriyanum* and *P. canhii* into the same group. The phylogenetic analyses using multiple genes would be helpful in the treatment of systematic position at subgenus/section levels. For the study on the taxonomic position of *P. rungsuriyanum*, the present phylogenetic analyses are primarily conducted based on the molecular dataset published by Guo et al. (2015). The results are consistent with the previous molecular studies (Chochai et al. 2012; Gorniak et al. 2014), indicating that the well-supported division of the genus *Paphiopedilum* into three subgenera *Parvisepalum*, *Brachypetalum* and *Paphiopedilum*.

In this study and the previous report by Gorniak et al. (2014), the positions of *P. rungsuriyanum* and *P. canhii* are discordant between plastid and nuclear gene trees. On the ITS-based tree (Additional file 4: Figure S1), *P. rungsuriyanum* is sister to species of the section *Paphiopedilum* (PP = 0.91), while *P. canhii* is sister to a clade comprising species of the subgenus *Brachypetalum* and section *Barbata*, but without bootstrap support. In the present phylogenetic analyses, *P. rungsuriyanum* is grouped with *P. canhii* in the same lineage (PP = 1.00) based on the *ACO* tree (Additional file 5: Figure S2). On the *DEF*4-based tree (Additional file 6: Figure S3), *P. rungsuriyanum* and *P. canhii* are embedded in the section *Paphiopedilum* (BP = 84; PP = 0.99). On the *RAD*51-based tree, *P. canhii* is embedded in the section *Paphiopedilum*, while *P. rungsuriyanum* is sister to the clade comprising species of sections *Paphiopedilum*, *Barbata*, *Coryopedilum* and *Pardalopetalum* (Additional file 7: Figure S4). Based on the plastid tree (Additional file 8: Figure S5), *P. rungsuriyanum* is embedded in the clade comprising sections *Barbata* and *Paphiopedilum*, while *P. canhii* is sister to the subgenus *Paphiopedilum*. According to the analysis from combined data (Fig. 5), both *P. rungsuriyanum* and *P. canhii* are sister to the section *Paphiopedilum* and embedded in the subgenus *Paphiopedilum*. The incongruence between plastid and nuclear gene trees may be caused by horizontal gene transfer, hybridization, and/or incomplete lineage sorting (Nishimoto et al. 2003; Maddison and Knowles 2006; Kim and Donoghue 2008; Petit and Excoffier 2009; Yu et al. 2013). In *Paphiopedilum*, based on the multiple low-copy nuclear genes and the network analyses, subgenus *Paphiopedilum* (particularly sections *Barbata*, *Cochlopetalum* and *Paphiopedilum*) had a higher species diversification rate than the other subgenera of *Paphiopedilum*, suggesting that hybridization plays an important role in speciation (Guo et al. 2015). Due to the lack of strong interspecific reproductive barriers in *Paphiopedilum* species, it is proposed that as the geographic and ecological changes (e.g. sea-level fluctuations) disrupted the species boundaries, the interspecific hybridization may lead to the genome introgression across species barriers and contribute to the reticulate evolution in *Paphiopedilum* (Guo et al. 2015).

In *P. canhii* and *P. rungsuriyanum*, their miniature plants with marbled leaves can be easily allied to the taxonomic position associated with species of the sections *Parvisepalum* (subgenus *Parvisepalum*) and *Barbata* (subgenus *Paphiopedilum*) as suggested by Averyanov et al. (2010). However, from molecular analyses, it is hard to connect *P. rungsuriyanum* to any species of subgenus *Parvisepalum*. Species of subgenus *Parvisepalum* have markedly different floral morphology from those of *P. rungsuriyanum*, such as the staminode without any umbo, the mammillated stigmatic surface and the granular pollinia (Additional file 9: Table S4). In *Paphiopedilum*, the staminode morphology provides taxonomically important information for species delimitation (Braem 1988; Cribb 1998). Morphologically, the staminode of *P. rungsuriyanum* looks intermediate between those of sections *Barbata* and *Paphiopedilum*, being half-moon shaped with three lobes and a slight umbo in the middle (Fig. 1c). The staminode of section *Barbata* is characterized by semi-lunate shape and more or less tri-lobed or tri-dentate, without any umbo. *P. rungsuriyanum* and species in section *Barbata* are alike in the staminode. Besides, as compared with other morphological characteristics, such as marbled leaves, single-flowered inflorescence and petal/sepal ratio, we may possibly suggest a close relation of this species with the section *Barbata* (Additional file 9: Table S4). Nevertheless, the close affinity to section *Barbata* (forming a clade with both sections *Barbata* and *Paphiopedilum*) is only revealed by the plastid analysis with weak support values (Additional file 8: Figure S5). In the analysis of combined data, *P. rungsuriyanum* is clustered with section *Paphiopedilum* species with high support values (Fig. 5). Although the floral morphology of *P. rungsuriyanum* does not resemble those of section *Paphiopedilum* species, it is noteworthy that section *Paphiopedilum* is characterized by staminode with a prominent umbo, and *P. rungsuriyanum* has a slight umbo in the middle of staminode as well. Guo et al. (2015) indicated that the sympatric distribution and the weak interspecific reproductive isolation may have facilitated the interspecific hybridization and led to higher diversification rate in subgenus *Paphiopedilum*. In *Paphiopedilum*, thousands of artificial interspecific hybrids have been made between species from different subgenera/sections and registered in the Royal Horticultural Society (http://apps.rhs.org.uk/horticulturaldatabase/orchidregister/orchidregister.asp), and we can observe various staminode morphologies in these artificial interspecific hybrids. Since Indochina is the hotspot of species in sections *Barbata* and *Paphiopedilum*, the intermediate staminode morphology of *P. rungsuriyanum* might be the results from introgression between sections of subgenus *Paphiopedilum* in the process of hybrid speciation.

## Conclusion

*Paphiopedilum rungsuriyanum* is characterized by the miniature plants with tessellated leaves, a single-flowered inflorescence, a flower having a helmet shaped lip with a V-shaped neckline, and a semi-lunate staminode with an umbo and tri-dents (Figs. 1, 2). These features distinguish *P. rungsuriyanum* from all of the other known sections/subgenera of *Paphiopedilum*. The subgenus *Paphiopedilum* forms a monophyletic group based on the combined analysis, and both *P. rungsuriyanum* as well as *P. canhii* are embedded in this clade. Moreover, in *P. rungsuriyanum* and *P. canhii*, the comparative studies on karyomorphology and the patterns of rDNA FISH also suggest a closer relationship to subgenus *Paphiopedilum*. At the present time, based on its specific morphological traits, we propose a new section *Laosianum* under the subgenus *Paphiopedilum* to accommodate *P. rungsuriyanum*, and describe it below. Furthermore, since *P. canhii* is also embedded in the subgenus *Paphiopedilum*, we suggest to change the status of subgenus *Megastaminodium* to section *Megastaminodium* under the subgenus *Paphiopedilum*.

## Taxonomic treatment

The new classification should be as follows:

Genus: Paphiopedilum

Subgenus: Paphiopedilum

Section Laosianum Lee, Chung, Sydara, Souliya & Luang Aphay, sect. nov.

Type: *Paphiopedilum rungsuriyanum* O. Gruss, N. Rungruang, Y. Chaisuriyakul et I. Dionisio.

### Etymology

The sectional name alludes to Laos, the name of the country where *P. rungsuriyanum* was found.

### Diagnosis

Although the *P. rungsuriyanum* and *P. canhii* have similar tessellated leaves, their flower morphologies are different from each other. The new remarkable section is distinct from other known subgenera/sections in the genus *Paphiopedilum* by possessing tessellated leaves, the oblong oval petals with intensive red–purple veins, the helmet shaped lip with a V-shaped neckline in the front, and the semi-lunate staminodial shield with trident at the base.

## Description

This is a monotypic section containing only *P. rungsuriyanum*. The section is characterized by its single-flowered inflorescence and the miniature plant with tessellated leaves. Although both of *P. rungsuriyanum* and *P. canhii* have miniature plants with tessellated leaves, there is a great difference between their flower morphologies. The lip is helmet shaped with incurved lateral lobes and a V-shaped neckline, and the petal is oval shape and intensively red–purple veins. *P. rungsuriyanum* has a semi-lunate staminode with an umbo and tri-dents that looks an intermediate morphology between those in sections *Barbata* and *Paphiopedilum*. The chromosome number of *P. rungsuriyanum* is 2n = 26.

Section Megastaminodium (Braem & O. Gruss) Lee, Chung, Sydara, Souliya & Luang Aphay, stat. nov.—Type: *Paphiopedilum canhii* Aver. & O. Gruss.

## Additional files

**Additional file 1: Table S1.** Voucher and GenBank accession number of plant materials used in this study. An asterisk (*) denotes the sequences of species that were obtained from GenBank.

**Additional file 2: Table S2.** Primers used in this study.

**Additional file 3: Table S3.** Results of the best fitting models from MrModel test for datasets.

**Additional file 4: Figure S1.** One of the most parsimonious trees from the analysis of ITS for *Paphiopedilum*. Bootstrap percentages (BP) >70 and Bayesian posterior probabilities (PP) are given for supported clades above the branches.

**Additional file 5: Figure S2.** One of the most parsimonious trees from the analysis of low-copy nuclear gene, *ACO* for *Paphiopedilum*. Bootstrap percentages (BP) >70 and Bayesian posterior probabilities (PP) are given for supported clades above the branches.

**Additional file 6: Figure S3.** One of the most parsimonious trees from the analysis of low-copy nuclear gene, *DEF*4 for *Paphiopedilum*. Bootstrap percentages (BP) >70 and Bayesian posterior probabilities (PP) are given for supported clades above the branches.

**Additional file 7: Figure S4.** One of the most parsimonious trees from the analysis of low-copy nuclear gene, *RAD*51 for *Paphiopedilum*. Bootstrap percentages (BP) >70 and Bayesian posterior probabilities (PP) are given for supported clades above the branches.

**Additional file 8: Figure S5.** One of the most parsimonious trees from the combined analysis of four plastid regions (*atp*I-*atp*H, *matK*, *trn*S-*trn*fM and *ycf*1) for *Paphiopedilum*. Bootstrap percentages (BP) >70 and Bayesian posterior probabilities (PP) are given for supported clades above the branches.

**Additional file 9: Table S4.** The comparison of main significant traits between subgenera and sections of *Paphiopedilum* by Gorniak et al. (2014) and the present study.
