# Supplementary material for: Taxonomic placement of Paphiopedilum rungsuriyanum (Cypripedioideae; Orchidaceae) based on morphological, cytological and molecular analyses
Source: Bot Stud. 2017 Mar 29;58:16. doi: 10.1186/s40529-017-0170-1 (PMC5432934; doi:10.1186/s40529-017-0170-1)
Supplement: Supplementary file 1 — Additional file 1: Table S1. Voucher and GenBank accession number of plant materials used in this study. An asterisk (*) denotes the sequences of species that were obtained from GenBank. [file 40529_2017_170_MOESM1_ESM.docx]

Table S1. Voucher and GenBank accession number of plant materials used in this study.

| Taxon | Vouchers/source | *atp*I-*atp*H | *mat*K | *ycf*1 | *trn*S-*trn*fM | ITS | *ACO* | *DEF4* | *RAD51* |
| --- | --- | --- | --- | --- | --- | --- | --- | --- | --- |
| Subgenus *Parvisepalum* |  |  |  |  |  |  |  |  |  |
| *P. armeniacum** | 2471/ NOCC | KP312208 | KP311994 | KP311695 | KP312321 | JQ660882 | KP312432 | KP312673 | KP312898 |
| *P. delenatii** | 1951/ NOCC | KP312209 | JQ182193 | JQ182265 | KP312322 | JQ660881 | KP312433 | KP312674 | KP312900 |
| *P. hangianum** | 2008/ NOCC | KP312214 | KP311999 | KP311700 | KP312326 | JX088558 | KP312437 | KP312678 | KP312907 |
| *P. malipoense** | 2316/ NOCC | KP312211 | KP311996 | KP311697 | KP312324 | JQ929336 | KP312434 | KP312676 | KP312902 |
| *P. micranthum** | 1919/ NOCC | KP312212 | KP311997 | KP311698 | KP312325 | JQ929338 | KP312436 | KP312677 | KP312903 |
| *P. vietnamense** | 2090/ NOCC | KP312216 | JQ182194 | JQ182266 | KP312329 | AY643433 | JQ182155 | KP312680 | KP312909 |
| Subgenus *Brachypetalum* |  |  |  |  |  |  |  |  |  |
| *P. bellatulum** | NOCC | KP312217 | JN181448 | JN181516 | KP312330 | JX088553 | KP312440 | KP312681 | KP312910 |
| *P*. *concolor** | 2511/ NOCC | KP312220 | KP312003 | KP311704 | KP312333 | JQ660878 | KP312441 | KP312684 | KP312915 |
| *P*. *niveum** | 6328/ NOCC | KP312226 | KP312009 | KP311710 | KP312339 | JQ660879 | KP312453 | KP312692 | KP312922 |
| *P*. *wenshanense** | 4548/ NOCC | KP312227 | KP312010 | KP311711 | KP312340 | JQ904599 | KP312454 | KP312693 | KP312923 |
| Subgeus *Paphiopedilum* |  |  |  |  |  |  |  |  |  |
| Section *Coryopedilum* |  |  |  |  |  |  |  |  |  |
| *P. gigantifolium** | 2799/ NOCC | KP312229 | KP312011 | KP311712 | KP312342 | JQ660879 | KP312455 | KP312695 | KP312925 |
| *P. glanduliferum** | 6182/ NOCC | KP312230 | KP312012 | KP311713 | KP312343 | JQ660864 | KP312457 | KP312697 | KP312927 |
| *P. kolopakingii** | 6321/ NOCC | KP312231 | KP312013 | KP311714 | KP312344 | JQ929331 | KP312458 | KP312699 | KP312929 |
| *P. philippinense** | 2035/ NOCC | KP312232 | KP312014 | KP311715 | KP312345 | JQ929341 | KP312459 | KP312700 | KP312930 |
| *P. rothschildianum** | 6071/ NOCC | KP312235 | KP312017 | KP311718 | KP312348 | JQ660865 | KP312463 | KP312703 | KP312933 |
| *P. stonei** | 022211-1/MBG | KP312237 | KP312019 | KP311720 | KP312350 | JQ660866 | KP312466 | KP312704 | KP312935 |
| *P. wilhelminae** | 6178/ NOCC | KP312239 | KP312021 | KP311722 | KP312352 | JQ929357 | KP312468 | KP312706 | KP312937 |
| Section *Pardalopetalum* |  |  |  |  |  |  |  |  |  |
| *P. dianthum** | 2104/ NOCC | KP312240 | JQ182192 | JQ182264 | KP312353 | JQ929315 | JQ182153 | KP312707 | KP312938 |
| *P. haynaldianum** | 2014/ NOCC | KP312242 | KP312023 | KP311724 | KP312355 | JQ929325 | KP312469 | KP312709 | KP312940 |
| *P. lowii** | 4624/ NOCC | KP312244 | KP312025 | KP311726 | KP312357 | JQ660867 | KP312471 | KP312711 | KP312942 |
| *P. parishii** | 2628/ NOCC | KP312245 | KP312026 | KP311727 | KP312358 | JQ660868 | KP312472 | KP312712 | KP312944 |
| Section *Cochlopetalum* |  |  |  |  |  |  |  |  |  |
| *P. glaucophyllum** | 002125-2/MBG | KP312246 | KP312027 | KP311728 | KP312359 | JQ929321 | KP312473 | KP312713 | KP312945 |
| *P. liemianum** | 6316/ NOCC | KP312248 | KP312029 | KP311730 | KP312361 | JQ929333 | KP312475 | KP312716 | KP312948 |
| *P. primulinum** | 011090-1/MBG | KP312249 | KP312030 | KP311731 | KP312362 | JQ929342 | KP312476 | KP312717 | KP312949 |
| *P. victoria-regina** | 921150-1/MBG | KP312252 | KP312032 | KP311733 | KP312365 | JQ929353 | KP312480 | KP312721 | KP312953 |
| Section *Paphiopedilum* |  |  |  |  |  |  |  |  |  |
| *P. barbigerum** | 1798/ NOCC | KP312254 | KP312035 | KP311736 | KP312368 | AY643442 | KP312484 | KP312724 | KP312957 |
| *P. charlesworthii** | 840166-1/MBG | KP312256 | KP312036 | KP311737 | KP312369 | JQ929310 | KP312485 | KP312725 | KP312959 |
| *P. druryi** | 913218-1/MBG | KP312258 | KP312038 | KP311739 | KP312371 | JQ660871 | KP312488 | KP312727 | KP312963 |
| *P. gratrixianum** | 3078/ NOCC | KP312262 | KP312042 | KP311743 | KP312375 | JQ660876 | KP312492 | KP312730 | KP312966 |
| *P. helenae** | 1820/ NOCC | KP312264 | KP312044 | KP311745 | KP312377 | JQ660877 | KP312495 | KP312733 | KP312969 |
| *P. insigne** | 962575-1/MBG | KP312270 | KP312049 | KP311750 | KP312383 | JQ660874 | KP312503 | KP312739 | KP312976 |
| *P. tigrinum** | 002233-3/MBG | KP312253 | KP312033 | KP311734 | KP312366 | JQ929351 | KP312482 | KP312722 | KP312954 |
| *P. villosum** | 913229-1/MBG | KP312278 | KP312057 | KP311758 | KP312390 | JQ660875 | KP312515 | KP312748 | KP312987 |
| Section *Barbata* |  |  |  |  |  |  |  |  |  |
| *P. acmodontum** | 2298/ NOCC | KP312279 | KP312058 | KP311759 | KP312391 | EF156081 | KP312517 | KP312749 | KP312989 |
| *P. barbatum** | 6320/ NOCC | KP312281 | KP312060 | KP311761 | KP312393 | JQ660872 | KP312519 | KP312750 | KP312990 |
| *P. bullenianum** | 2057/ NOCC | KP312282 | KP312061 | KP311762 | KP312394 | KC692110 | KP312521 | KP312751 | KP312991 |
| *P. callosum** | 25191/ NOCC | KP312283 | KP312062 | KP311763 | KP312395 | JQ929308 | KP312523 | KP312752 | KP312993 |
| *P. ciliolare** | 2083/ NOCC | KP312285 | KP312064 | KP311765 | KP312397 | JQ929311 | KP312525 | KP312754 | KP312995 |
| *P. lawrenceanum** | 882380-1/MBG | KP312290 | KP312069 | KP311770 | KP312401 | JQ929332 | KP312529 | KP312757 | KP312999 |
| *P. mastersianum** | 4614/ NOCC | KP312291 | KP312070 | KP311771 | KP312402 | JQ929337 | KP312531 | KP312758 | KP313000 |
| *P. purpuratum** | 1615/ NOCC | KP312293 | KP312072 | KP311773 | KP312404 | EF156131 | KP312533 | KP312760 | KP313002 |
| *P. superbiens** | 2142/ NOCC | KP312298 | KP312077 | KP311778 | KP312409 | JQ929350 | KP312538 | KP312765 | KP313007 |
| *P. tonsum** | 822999-2/MBG | KP312299 | KP312078 | KP311779 | KP312410 | JQ929352 | KP312539 | KP312766 | KP313008 |
| *P. venustum** | 013228-1/MBG | KP312302 | KP312081 | KP311782 | KP312413 | HQ998475 | KP312542 | KP312770 | KP313011 |
| *P. violascens** | 6323/ NOCC | KP312304 | KP312083 | KP311784 | KP312415 | JQ929355 | KP312544 | KP312773 | KP313013 |
| *P. wardii** | 2701/ NOCC | KP312305 | JN181450 | JN181518 | KP312416 | JQ929356 | JN181404 | KP312774 | KP313014 |
| Section *Megastaminodium* |  |  |  |  |  |  |  |  |  |
| *P. canhii* | Yung-I Lee201505 /TNM | KP312314 | KP312092 | KP311793 | KP312425 | JQ660880 | registering | registering | registering |
| Section *Laosianum* |  |  |  |  |  |  |  |  |  |
| *P. rungsuriyanum* | Yung-I Lee201504 /TNM | registering | KX609596* | registering | registering | KX609594* | registering | registering | registering |
| Outgroups |  |  |  |  |  |  |  |  |  |
| *Mexipedium xerophyticum** | 902725-5/MBG | KP312320 | JN181455 | JN181523 | KP312431 | JQ660883 | JN181412 | — | KP313035 |
| *Phragmipedium longifolium** | 840941-1/MBG | KP312319 | JN181454 | JN181522 | KP312430 | JQ929359 | JN181410 | — | KP313033 |

An asterisk (*) denotes the sequences of species that were obtained from GenBank

Dashes indicate unavailable sequence

MBG: Missouri Botanical Garden

NOCC: The National Orchid Conservation Center (The Orchid Conservation & Research Center of Shenzhen)

TNM: Herbarium of the National Museum of Natural Science
