# Supplementary material for: Taxonomic placement of Paphiopedilum rungsuriyanum (Cypripedioideae; Orchidaceae) based on morphological, cytological and molecular analyses
Source: Bot Stud. 2017 Mar 29;58:16. doi: 10.1186/s40529-017-0170-1 (PMC5432934; doi:10.1186/s40529-017-0170-1)
Supplement: Supplementary file 2 — Additional file 2: Table S2. Primers used in this study. [file 40529_2017_170_MOESM2_ESM.docx]

Table S2. Primers used in this study.

| Regions | Primers | Primer sequences (5'-3') | References |
| --- | --- | --- | --- |
| *atp*I-*atp*H | *atp*I | TATTTACAAGYGGTATTCAAGCT | Shaw et al. 2007 |
|  | *atp*H | CCAAYCCAGCAGCAATAAC | Shaw et al. 2007 |
| *mat*K | 390F | CGATCTATTCATTCAATATTTC | Cuénoud et al. 2002 |
|  | 1326R | TCTAGCACACGAAAGTCGAAGT | Cuénoud et al. 2002 |
| *trn*S-*trn*fM | *trn*S(UGA) | GAGAGAGAGGGATTCGAACC | Demesure et al. 1995 |
|  | *trn*fM(CAU) | CATAACCTTGAGGTCACGGG | Demesure et al. 1995 |
| *ycf*1 | *ycf*1_3720F | TACGTATGTAATGAACGAATGG | Neubig et al. 2009 |
|  | *ycf*1_5500R | GCTGTTATTGGCATCAAACCAATAGCG | Neubig et al. 2009 |
| ITS | 101AB | ACGAATTCATGGTCCGGTGAAGTGTTCG | Douzery et al. 1999 |
|  | 102AB | GAATTCCCCGGTTCGCTCGCCGTTAC | Douzery et al. 1999 |
| *ACO* | *ACO*E1aF | GCNTGYGAGAACTGGGGHTTCTTYGAG | Guo et al. 2012 |
|  | *ACO*E2aR | ATGGTCTTCATGGCCTCAAACCT | Guo et al. 2012 |
| *DEF*4 | *DEF*4E1bF | AGGGGAAAGATTGAGATAAAG | Guo et al. 2015 |
|  | *DEF*4aR | TTCTTCTTGTAGGTGTCAGTC | Guo et al. 2015 |
| *RAD*51 | *RAD*51E5F | AGAGGGHACATTCAGACCACA | Guo et al. 2015 |
|  | *RAD*51E8bR | CCATCTACTTGTGCGACAACTTG | Guo et al. 2015 |

References

Shaw J, Lickey EB, Schilling EE, Small RL. Comparison of whole chloroplast genome sequences to choose noncoding regions for phylogenetic studies in angiosperms: the tortoise and the hare III. American Journal of Botany. 2007; 94, 275-288.

**C**uénoud P, Savolainen V, Chatrou LW, Powell M, Grayer RJ, Chase MW. Molecular phylogenetics of Caryophyllales based on nuclear 18S rDNA and plastid *rbc*L, *atp*B, and *mat*K DNA sequences. American Journal of Botany. 2002; 89, 132-144.

Demesure B, Sodzi N, Petit RJ. A set of universal primers for amplification of polymorphic noncoding regions of mitochondrial and chloroplast DNA in plants. Molecular Ecology. 1995; 4, 129-131.

Neubig KM, Whitten WM, Carlsward BS, Blanco MA, Endara L, et al. Phylogenetic utility of *ycf*1 in orchids: a plastid gene more variable than *mat*K. Plant Systematics and Evolution. 2009; 277, 75-84.

Douzery EJP, Pridgeon AM, Kores P, Linder HP, Kurzweil H, Chase MW. Molecular phylogenetics of *Disease* (Orchidaceae): a contribution from nuclear ribosomal ITS sequences. American Journal of Botany. 1999; 86: 887-899.

Guo YY, Luo YB, Liu ZJ, Wang XQ. Evolution and biogeography of the slipper orchids: Eocene vicariance of the conduplicate genera in the Old and New World tropics. PLoS ONE. 2012; 7, e38788.

Guo YY, Luo YB, Liu ZJ, Wang XQ. Reticulate evolution and sea-level fluctuations together drove species diversification of slipper orchids (*Paphiopedilum*) in South-East Asia. Molecular Ecology. 2015; 24: 2838-2855.
