# Supplementary material for: Taxonomic placement of Paphiopedilum rungsuriyanum (Cypripedioideae; Orchidaceae) based on morphological, cytological and molecular analyses
Source: Bot Stud. 2017 Mar 29;58:16. doi: 10.1186/s40529-017-0170-1 (PMC5432934; doi:10.1186/s40529-017-0170-1)
Supplement: Supplementary file 3 — Additional file 3: Table S3. Results of the best fitting models from MrModel test for datasets. [file 40529_2017_170_MOESM3_ESM.docx]

Table S3. Results of the best fitting models from MrModel test for datasets.

| Datasets | The best fitting models |
| --- | --- |
| combined cpDNA | GTR+I+G |
| ITS | GTR+I+G |
| *ACO* | SYM+I+G |
| *DEF*4 | HKY+G |
| *RAD*51 | GTR+G |
