# Supplementary figures and images for: Taxonomic placement of Paphiopedilum rungsuriyanum (Cypripedioideae; Orchidaceae) based on morphological, cytological and molecular analyses
Source: Bot Stud. 2017 Mar 29;58:16. doi: 10.1186/s40529-017-0170-1 (PMC5432934; doi:10.1186/s40529-017-0170-1)

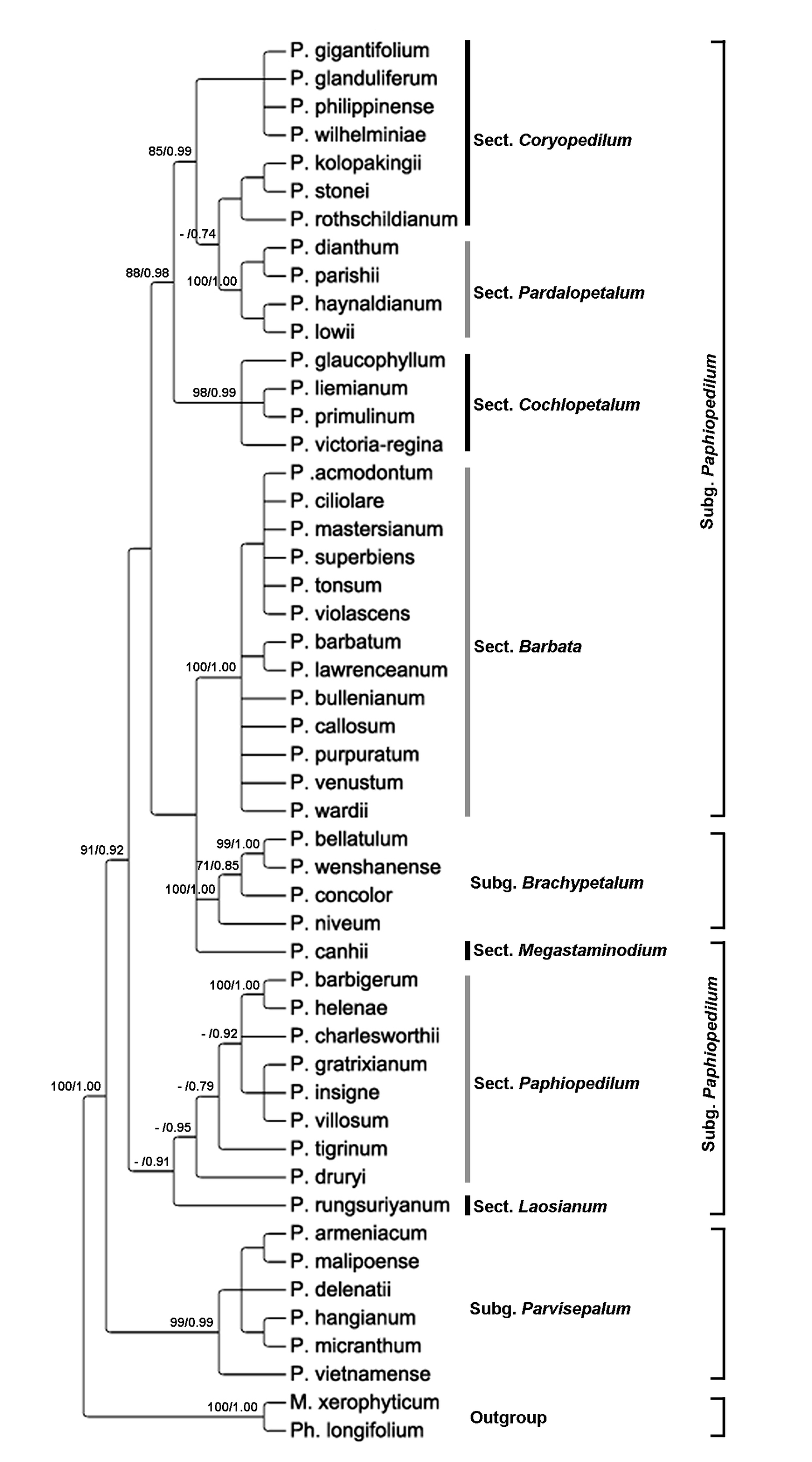

Supplement: Supplementary file 4 — Additional file 4: Figure S1. One of the most parsimonious trees from the analysis of ITS for Paphiopedilum. Bootstrap percentages (BP) >70 and Bayesian posterior probabilities (PP) are given for supported clades above the branches. [file 40529_2017_170_MOESM4_ESM.tif]

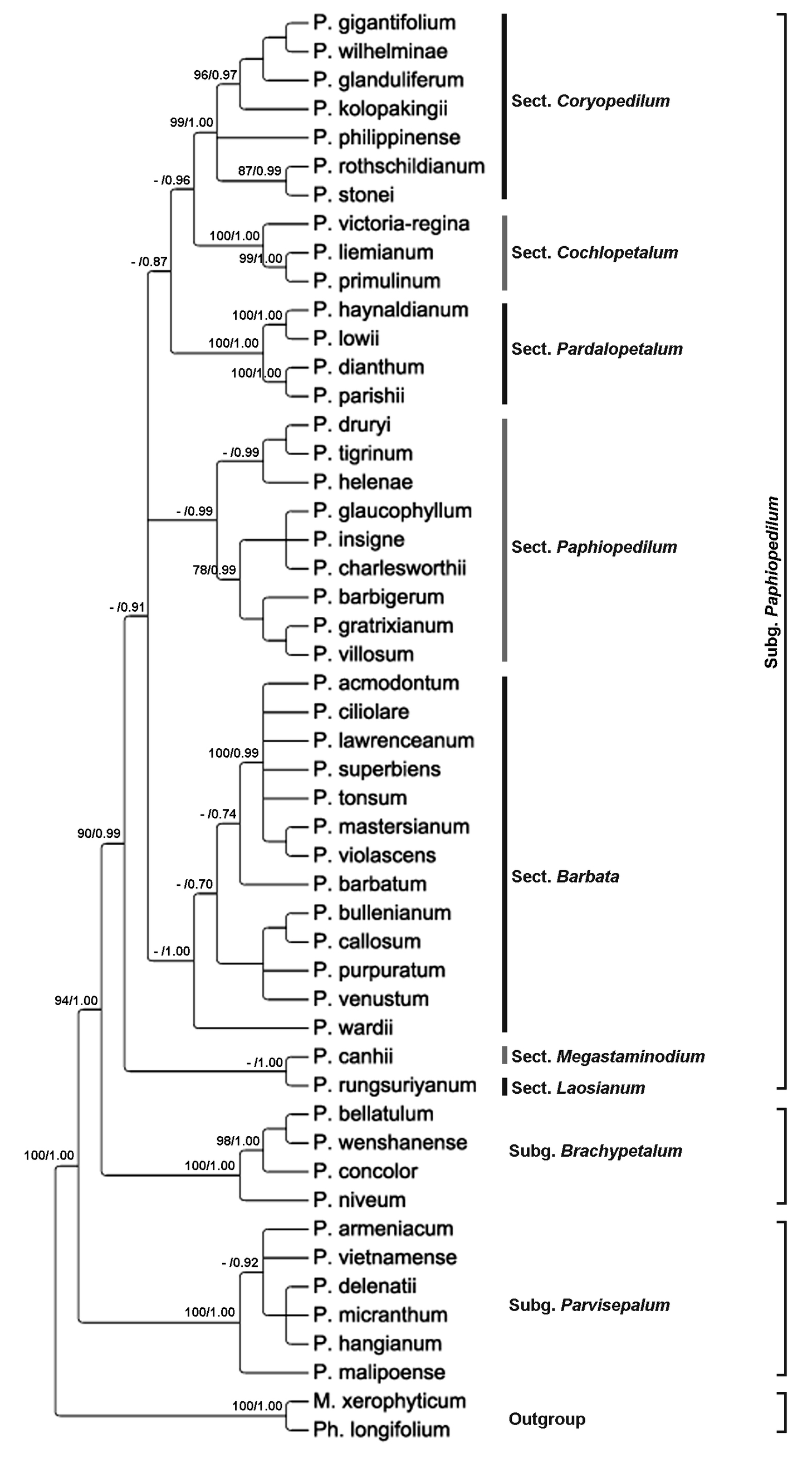

Supplement: Supplementary file 5 — Additional file 5: Figure S2. One of the most parsimonious trees from the analysis of low-copy nuclear gene, ACO for Paphiopedilum. Bootstrap percentages (BP) >70 and Bayesian posterior probabilities (PP) are given for supported clades above the branches. [file 40529_2017_170_MOESM5_ESM.tif]

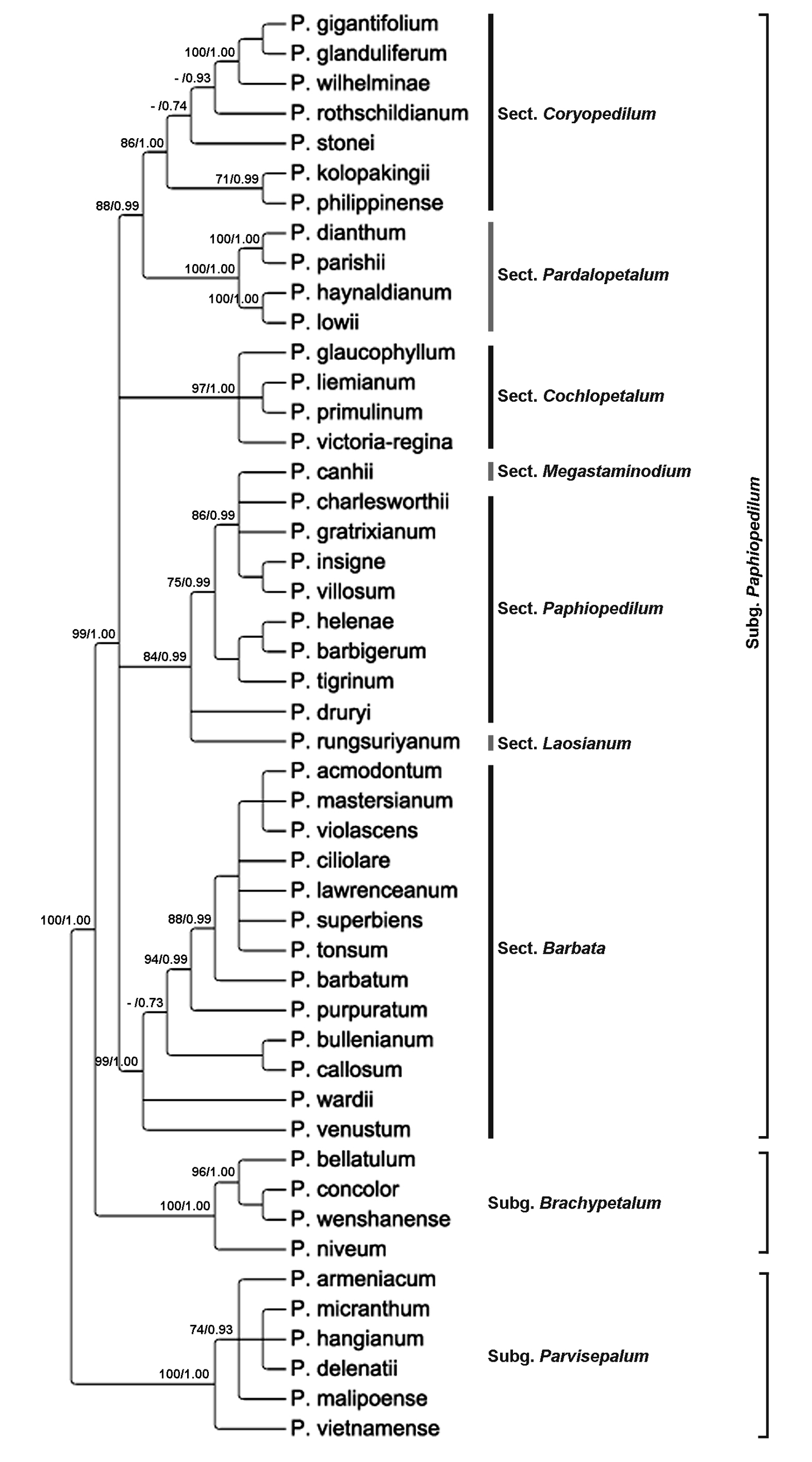

Supplement: Supplementary file 6 — Additional file 6: Figure S3. One of the most parsimonious trees from the analysis of low-copy nuclear gene, DEF4 for Paphiopedilum. Bootstrap percentages (BP) >70 and Bayesian posterior probabilities (PP) are given for supported clades above the branches. [file 40529_2017_170_MOESM6_ESM.tif]

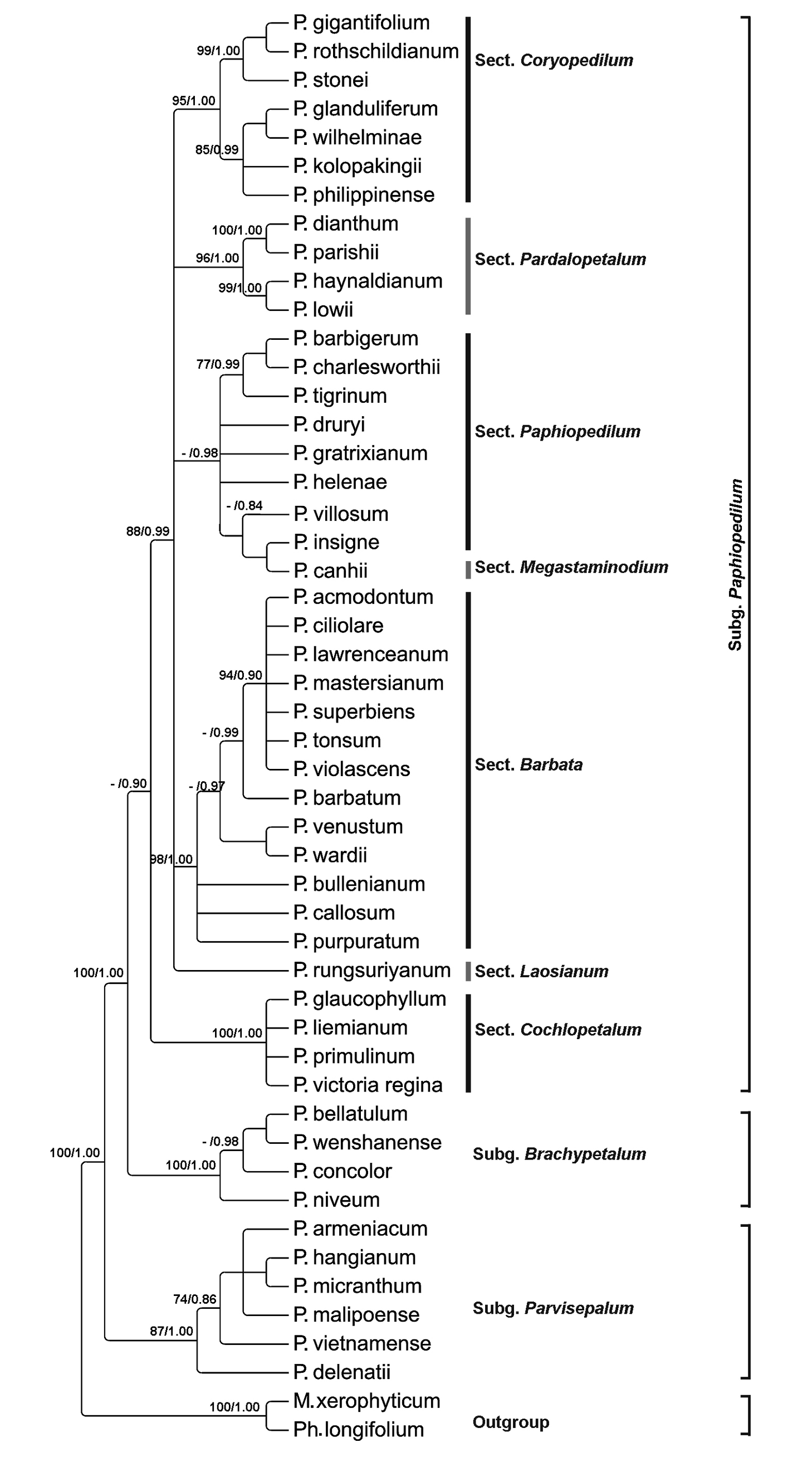

Supplement: Supplementary file 7 — Additional file 7: Figure S4. One of the most parsimonious trees from the analysis of low-copy nuclear gene, RAD51 for Paphiopedilum. Bootstrap percentages (BP) >70 and Bayesian posterior probabilities (PP) are given for supported clades above the branches. [file 40529_2017_170_MOESM7_ESM.tif]

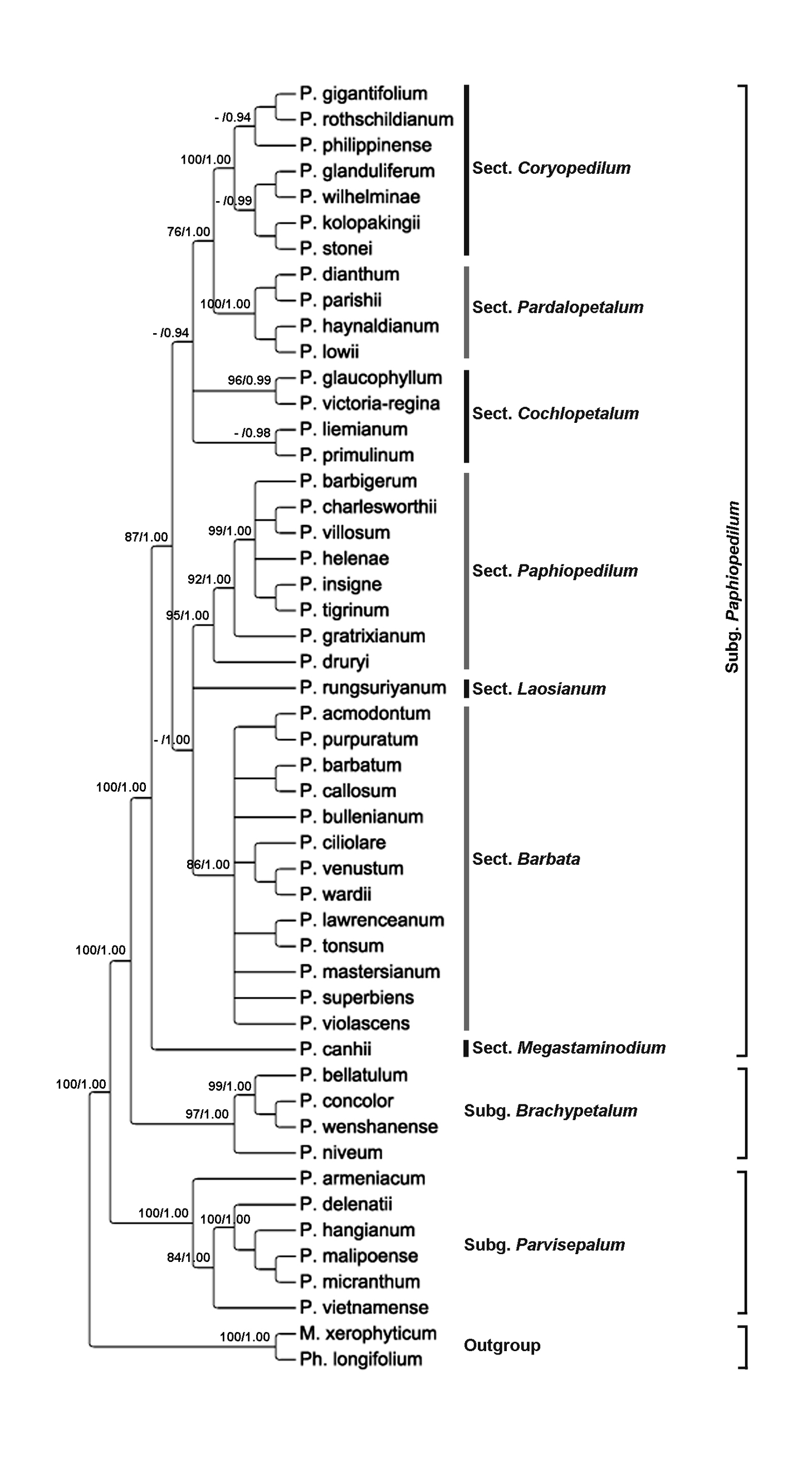

Supplement: Supplementary file 8 — Additional file 8: Figure S5. One of the most parsimonious trees from the combined analysis of four plastid regions (atpI-atpH, matK, trnS-trnfM and ycf1) for Paphiopedilum. Bootstrap percentages (BP) >70 and Bayesian posterior probabilities (PP) are given for supported clades above the branches. [file 40529_2017_170_MOESM8_ESM.tif]
