# Supplementary material for: Taxonomic placement of Paphiopedilum rungsuriyanum (Cypripedioideae; Orchidaceae) based on morphological, cytological and molecular analyses
Source: Bot Stud. 2017 Mar 29;58:16. doi: 10.1186/s40529-017-0170-1 (PMC5432934; doi:10.1186/s40529-017-0170-1)
Supplement: Supplementary file 9 — Additional file 9: Table S4. The comparison of main significant traits between subgenera and sections of Paphiopedilum by Gorniak et al. (2014) and the present study. [file 40529_2017_170_MOESM9_ESM.docx]

Table S4. The comparison of main significant traits between subgenera and sections of *Paphiopedilum* by Gorniak et al. (2014) and the present study.

| Traits | Parvisepalum | Brachypetalum |  |  | Paphiopedilum |  |  |  |  |
| --- | --- | --- | --- | --- | --- | --- | --- | --- | --- |
|  |  |  | Laosianum^*^ | Megastaminodium | Paphiopedilum | Barbata | Coryopedilum | Pardalopetalum | Cochlopetalum |
| Staminodium shape and lobation | Subrobicular,subquadrate,obovate, triangularovate | Subquadrate, rhombic, apically 3-lobed, the middle lobe the longest | Semi-lunate,3-dentate, the middle lobe the longest | Oblong-ovate, very large | Ovate, elliptic, transversely elliptic, shortly 3-dentate at apex | Semi-lunate, 3-dentate, the middle lobe the shortest | Subquadrate to digitate, occasionally bifid apically | Ovate, oblong-ovate, ovate-triangular, 2- or 3-lobed at apex | Ovate, elliptic, shortly apiculate |
| Staminodium umbo | No | No | Present | No | Usually present | No | No | No | No |
| Staminodium/stigma ratio | Longer than stigma | Subequal to stigma | Subequal | Longer than stigma | Subequal | Shorter | Shorter than or equal to stigma | Subequal | Subequal |
| Stigmatic surface | Mammillate | Smooth | Smooth | Smooth | Smooth | Smooth | Smooth | Smooth | Smooth |
| Pollinia | Granulous | Viscid | Viscid | Viscid | Viscid | Viscid | Viscid | Viscid | Viscid |
| Lip shape | Subsessile, inflated | Subsessile, laterally  compressed | Tubular, apically upcurved and inflated | Tubular, apically upcurved and inflated | Basally narrow, tubular, short, inflated above | Tubular, apically upcurved and inflated | Basally narrow, tubular, apically upcurved and inflated | Basally narrow, tubular, apically upcurved and inflated | Basally narrow, tubular, short, much inflated above |
| Lip margins | All incurved | All incurved | Basal incurved, apical part erect without auricles | Basal incurved, apical part erect without auricles | Basal incurved, apical part erect with auricles | Basal incurved, apical part erect with auricles | Basal incurved, apical part erect without auricles | Basal incurved, apical part erect with auricles | Basal incurved, apical part erect with auricles |
| Lip lateral lobes | No | Prominent | Prominent | Prominent | Prominent | Prominent, usually warted | Obscure | Prominent | Prominent |
| Flower number | One | One | One | One | One | One | Multi-flowered | Multi-flowered | Multi-flowered |
| Inflorescence development | -- | -- | -- | -- | -- | -- | Simultaneously | Simultaneously | Successively |
| Petals/sepals ratio | Larger | Larger | Narrower | Narrower | Narrower | Narrower | Narrower | Narrower | Narrower |
| Leaf color | Marbled | Marbled | Marbled | Marbled | Plane green | Marbled | Plane green | Plane green | Usually plane green |

* Besides the data of section Laosianum from this study, the data of other subgenera and sections were obtained from Gorniak et al. (2014).
